# Identification of Novel Sources of Resistance to Seed Weevils (*Bruchus* spp.) in a Faba Bean Germplasm Collection

**DOI:** 10.3389/fpls.2018.01914

**Published:** 2019-01-09

**Authors:** Estefanía Carrillo-Perdomo, Blandine Raffiot, Damien Ollivier, Chrystel Deulvot, Jean-Bernard Magnin-Robert, Nadim Tayeh, Pascal Marget

**Affiliations:** ^1^Agroécologie, AgroSup Dijon, INRA, Université Bourgogne Franche-Comté, Dijon, France; ^2^Terres Inovia, Thierval-Grignon, France

**Keywords:** faba bean (*Vicia faba* L.), seed weevil resistance, *Bruchus* spp., seed infestation, larval development

## Abstract

Seed weevils (*Bruchus* spp.) are major pests of faba bean, causing yield losses, and affecting marketability. Our objective was to identify stable sources of resistance to seed weevil attacks, determine the climatic factors that most influenced its incidence and its relationship with some phenological and agronomic traits. The accessions “BOBICK ROD115,” “CÔTE D’OR,” “221516,” and “NOVA GRADISKA” showed increased resistance to penetration and development of larvae. Other accessions such as “QUASAR,” “109.669,” and “223303” exhibited resistance to larval development. The results of this work suggest the presence of different defense mechanisms to seed weevils in faba bean, which in the future could be introgressed in elite cultivars to create resistant varieties and contribute to more sustainable agriculture with less need for pesticides. The temperature, rainfall, and humidity seemed to be the climatic factors most influencing faba bean seed weevil attack while the precocity and the small weight of the seeds were correlated with lower infestation rates in the different experiments.

## Introduction

Faba bean (*Vicia faba* L.) contributes to meet the basic dietary needs of millions of people and animals around the world thanks to its high content of proteins, carbohydrates, dietary fibers, and micronutrients (Mulualem et al., 2012). It plays a crucial role in environmentally friendly agriculture due to its ability to improve soil fertility by fixing atmospheric nitrogen and increasing crop yields when used in crop rotation with cereals. Seed weevils of the genus *Bruchus*, hereinafter referred to as faba bean seed weevils (*Bruchus* spp.), are among the pests that produce the greatest losses in *V. faba* and that most affect the marketability of the grain (Kharrat et al., 2006; Maalouf et al., 2009). Although *B. rufimanus* Boh. is the seed weevil species most affecting *V. faba*, it has been reported that *B. dentipes* Baudi and *B. atomarius* L. also use *V. faba* as host plant (Kergoat et al., 2007). *B. rufimanus* is a univoltine species present in most of the regions where faba bean is grown (Hoffman et al., 1962; Hulme, 2009). Throughout winter and at the time of seedling emergence in the field, adults diapause by hiding under the bark of trees or lichens while larvae or pupae diapause in stored seeds (Tran et al., 1993). In spring, the males terminate the diapause when the photoperiod reaches 16 h per day (being 18 h of light/6 h darkness the optimum), the diurnal temperature reaches 20°C and faba bean pollen is available (Tran and Huignard, 1992; Roubinet, 2016). The females become reproductively active at the beginning of the pod-setting period and oviposit up to 10 eggs on the surface of each green pod. They are able to oviposit up to one hundred eggs while they are sexually active (Tran and Huignard, 1992; Medjdoub-Bensaad et al., 2015; Roubinet, 2016). Larvae emerge from the egg in about 10 days and then go through the pods and seeds. Up to three larvae can colonize a seed and develop for about 3 months while feeding on the cotyledons. Then, the larvae pupate and reach the adult stage. The adults emerge out of the seed through a circular perforation of the seed coat. Seed weevils decrease seed viability by damaging the embryo and spreading bacterial and fungal infections (Sallam, 2013). Moreover, the reduced reserves in the infested cotyledons can slow down plantlet growth and negatively impact the success of establishment of germinated seeds. Attacks by seed weevils also reduce grain weight and affect the colour, taste, smell and nutritional value of the grains (Christensen and Kaufmann, 1965; Sallam, 2013).

Nowadays, the management of faba bean seed weevils depends to a large extent on the use of chemical insecticides. However, a large number of them have been banned as they have a negative impact on the environment, humans and non-target organisms including pollinators. Post-harvest treatment is necessary to limit the emergence of adult weevils from the inside of the stored seeds and to comply with market requirements that prohibit the presence of live insects in the grains for export. Biological control has been attempted by using predators and parasitoids (Titouhi et al., 2017) or by applying plant essential oil treatments (Jemâa, 2014; Amzouar et al., 2016; Titouhi et al., 2017). In addition, agronomical and cultural practices have also been applied (Keneni et al., 2011; Mishra et al., 2018). Nonetheless, their effectiveness is limited and their use in large production areas implies a considerable economic investment.

In this context, breeding of resistant cultivars is the most appropriate approach to achieve durable and efficient levels of resistance that meet the requirements of the agri-food sector and promote sustainable agriculture. However, no resistant faba bean cultivars have been developed so far. Bruchid resistance has been studied in other legume crops where wild relatives are the main source of resistance (Aznar-Fernández et al., 2017; Mishra et al., 2018). Nonetheless, some sources of resistance have also been identified in cultivated species such as in *Vigna mungo* (L.) Hepper (black gram) (Dongre et al., 1996), *Cicer arietinum* L. (chickpea) (Athiepacheco et al., 1994; Shaheen et al., 2006), *Phaseolus vulgaris* L. (common bean) (Ishimoto and Chrispeels, 1996; Goossens et al., 2000), *Vigna unguiculata* (L.) Walp. (cowpea) (Redden et al., 1983; Adam and Baidoo, 2008), *V. radiata* (L.) R. Wilczek (mungbean) (Schafleitner et al., 2016), *Pisum sativum* L. (pea) (Morton et al., 2000; Clement et al., 2002), or *Cajanus cajan* (L.) Millsp. (pigeon pea) (Jadhav et al., 2012). In faba bean, no completely resistant accession has been identified so far (Seidenglanz and Huňady, 2016; Seidenglanz et al., 2017). The screening of germplasm collections with genetic diversity is thus necessary to successfully identify sources of resistance. In the present work, we have screened a faba bean germplasm collection with the aim of (1) identifying stable sources of resistance to seed weevils, (2) revealing the climatic factors most influencing the pest incidence, and (3) determining the relationship between the pest incidence and some phenological and agronomic traits.

## Materials and Methods

### Plant Material

**FIGURE 1:**
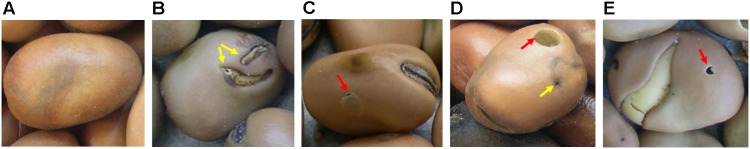
Healthy and *Bruchus* spp. infested faba bean seeds. **(A)** healthy seed; **(B)** surface damage caused by a seed weevil larvae (yellow arrows) that have fed on the external part of the cotyledon; **(C)** circular spot or window (red arrow) on the seed coat behind which there is still an adult of seed weevil; **(D)** infested seed presenting an emergence hole (red arrow) of an adult of seed weevil and the entry hole (yellow arrow) and path taken by a larvae to reach the cotyledons; **(E)** emergence hole (red arrow) of an adult of seed weevil parasitoid.

The faba bean germplasm collection (Duc et al., 2010) consisting of 1858 faba bean accessions available at the Institut National de la Recherche Agronomique (INRA) at Dijon (France) and including accessions from *V. faba* subsp. *faba* var. *equina* Pers., *V. faba* subsp. *faba* var. *minor Peterm*., *V. faba* subsp. *paucijuga* (Alef.), and *V. faba* subsp. *faba* var. *major* Harz. (synonym: *V. faba* var. *faba*) was screened in 2007 (Supplementary Table 1). It was decided to use a practical and fast approach to discard the most susceptible genotypes and maintain only the accessions that were less damaged by the bruchids. The experiments were conducted at the experimental farm of Epoisses in Bretenière (France) (Latitude 47°24′10″N; Longitude 5°11′40″E; Altitude 210 m). The percentage of healthy seeds (% HS) (Supplementary Figure 1 and Figure 1A) out of a set of 100 randomly selected seeds was visually quantified at each time for each accession. Accessions were then ranged accordingly into four groups: group 1, 0–25% of infested seeds; group 2, 26–50% of infested seeds; group 3, 51–75% of infested seeds; group 4, 76–100% of infested seeds (Supplementary Figure 1). A seed was considered infested if it showed: (a) a superficial damage caused by a larva that has passed through the seed coat and has even fed briefly on the outside of the cotyledon, but has not managed to reach the inside of the seed and complete its development (SD) (Figure 1B); (b) a circular “window” on the seed coat behind which there is an adult of faba bean seed weevil (Figure 1C); (c) a circular emergence hole caused by an adult bruchid (Figure 1D); (d) an emergence hole caused by adults of parasitoids that develop within the larvae of the seed weevil, including *Triaspis thoracicus* Curtis (braconide wasp), *Chremylus rubiginosus* Nees., and *Dinarmus acutus* Thomson (Roubinet, 2016) (Figure 1E).

In 2008, a field validation of a germplasm selection of 120 accessions that showed reduced infestation (less than 25% of infestation) (Supplementary Table 1) was carried out with a completely randomized design. Fifteen to 20 seeds by accession were sown in rows of 2.5 m with inter-row distance of 1 m. The semi-early spring variety “MÉLODIE” (*V. faba* subsp. *faba* var. equina) was included in the trial as a susceptible control. Since the experimental farm has a known history of high levels of seed weevil infestation, we relied on natural infestation. No pesticides were applied on the experimental plots or surrounding fields during the experiments. The trial was chemically weeded. The plants were harvested mechanically at maturity and threshed. The seeds were kept at room temperature for 1 month to favor a homogeneous development of the weevils prior to their storage at 4°C. One-hundred seeds were randomly selected for the quantification of the percentage of seed infestation for each accession as described above.

### Field Experiments and Assessments

A germplasm subset of 27 accessions with less than 25% of infestation was selected according to the description in “Plant Material” section (Supplementary Table 1). The cultivar “MÉLODIE” was selected as the moderately susceptible control and the traditional landrace “ILB 551” as the highly susceptible control (both selected from the first phenotyping step). These 29 accessions were evaluated in the experimental farm of Epoisses during the 2009 and 2010 growing seasons. Two sowings per year were carried out in each plot to assess the effects of sowing date on seed infestation. Sowings were conducted on February 15th and March 30th in 2009 and on March 3rd and April 12th in 2010. The field plot GPS coordinates were latitude 47°24′20″N and longitude 5°09′55″E in 2009 and latitude 47°24′10″N and longitude 5°09′01″E in 2010. Climatic parameters recorded by a nearby weather station are available in Table 1.

**Table 1:** Description of the four environments (defined as a combination of sowing date and year) where the 29 faba bean accessions selected after the pre-screening were evaluated.

Environment	Sowing date	Average date of first of flowering	Average date of last of flowering	Average date of first pod-setting	Tmax^a^ (°C)	Tmin^b^ (°C)	AvT^c^ (°C)	Thermal amplitude (°C)	Hmax^d^ (%)	Hmin^e^ (%)	AvH^f^ (%)	Rainfall (mm)	Imax rainfall^g^ (mm)	Smax wind^h^	AvS wind^i^	Global horizontal irradiance (J/m^2^)	Insolation duration (h)	D20T^j^	Dmax 20T^k^	Dmax 6W^k^
2009_S1	15/02	14/05	09/06	25/05	30.2	3.3	16.4	12.3	95.5	46.2	73.9	3.4	5.5	7	1.7	2186.8	8.9	2	27	26
2009_S2	30/03	26/05	20/06	04/06	30.2	5.2	16.9	12.1	95.8	45.8	74.1	3	5.4	7.3	1.8	2257.9	9.2	3	30	29
2010_S1	04/03	29/05	23/06	05/06	29.2	3.8	16.1	10.7	96.4	50.3	76.8	4.4	7.6	7	1.7	1880.5	6.9	2	25	30
2010_S2	12/04	16/06	10/07	26/06	33.5	7.1	19.8	12.4	94.3	44.7	71.8	3	4.7	4.7	1.5	2184.9	8.7	17	29	27


*^a^Tmax Maximum temperature. ^b^Tmin Minimum temperature. ^c^AvT Average temperature. ^d^Hmax Maximum relative humidity. ^e^Hmin Minimum relative humidity. ^f^AvH Average relative humidity. ^g^ImaxRainfall Maximum intensity of rainfall. ^h^SmaxWind Maximum speed of wind. ^i^AvSWind Average speed of wind. ^j^D20T Number of days with an average temperature above 20°C. ^k^Dmax20T number of days in which the maximum temperature has reached or exceeded 20°C. ^l^Dmax6W Number of days when the maximum wind speed is higher than 6 km/h.*

Each experimental assay consisted of three randomized blocks. In each block, 20 seeds were sown in two consecutive rows representing each accession. Intra and inter-row distances were 30 cm and 1 m, respectively. As in the pre-screening, no pesticides were applied and plots were chemically weeded. Infestation relied on natural occurrence. Plants were mechanically harvested at maturity and threshed. The seeds were kept at room temperature for 1 month to favor a homogeneous development of the weevils before storage at 4°C. One-hundred seeds per accession were randomly selected for the quantification of the percentage of healthy seeds (% HS) (Figure 1A) for which the percentage of surface damage (% SD) (Figure 1B) and emergence holes (% EH) (Figures 1C–E) were subtracted.

The days from sowing date to first and last flowering and first pod-setting (50% of the plants of the accession) (DFF, DLF, and DFP, respectively) in each environment were noted and the duration of flowering (DF) was calculated. All these traits were related to the semi-early flowering (Seidenglanz and Huňady, 2016) and moderately susceptible control “MÉLODIE” to evaluate the effect that precocity may have on the incidence of infestation. In addition, the color of the flowers (FC) (Supplementary Figure 2A) and seeds (SC) (Supplementary Figure 2B) were noted because they are related to the content of tannins in the seed (Cabrera and Martin, 1986). Furthermore, the thousand-seed weight (TSW) for each accession in each environment was also scored.

### Statistical Analysis

Analysis of variance (ANOVA) for a complete block randomized design was conducted using Statistix Version 9 with accession (G) and environment (E) as fixed factors in order to determine the genotypic (G) and the genotype × environment (G×E) interaction effects for seed weevil infestation in the studied *V. faba* accessions. Environments were defined as the combination of the year and the sowing date so each sowing date in a given year was considered as a separate environment. *F*-ratios were used to test the effects of the randomized complete block experiments combining year-sowing date environments (McIntosh, 1983).

An heritability-adjusted genotype main effect plus genotype–environment interaction (HA-GGE) biplot analysis was applied in order to eliminate the interactions between variables and to take into account the genotype and genotype × environment (GGE) interactions in the analysis (Yan and Holland, 2010). Singular value decomposition was achieved through symmetric scaling (scaling factor “*f”* = 0.5) since it bears most of the properties associated to other scaling methods (Yan and Rajcan, 2002) and “tester” centering. The analysis was carried out in R version 3.5.0 (package “GGEBiplots”).

To evaluate the influence of the different environmental factors (Table 1) on % HS, % EH, and % SD a canonical correspondence analysis (CCA) was carried out. The environmental factors included in the analysis were: minimum, maximum and average temperature (Tmin, Tmax, AvT); thermal amplitude (TermAmp); minimum, maximum, and average relative humidity (Hmin, Hmax, AvH); rainfall, maximum intensity of rain (ImaxRainfall), maximum, and average speed of the wind (SmaxWind, AvSWind), global horizontal irradiance (GlobIrrad) and insolation duration (InsolDur), number of days with an average temperature above 20°C (D20T), number of days in which the maximum temperature has reached or exceeded 20°C (Dmax20T), number of days when the maximum wind speed has reached or exceeded 6 km/h (Dmax6W). Climatic data from the beginning of flowering (first accession to bloom) to the beginning of pod-setting (last accession to bear pods) were obtained from the CLIMATIK portal of INRA. Analysis was carried out in R version 3.5.0 (package “vegan”).

ANOVA was conducted in order to test significant differences among accessions and sowing dates for DF, DFF, DLF, DFP, and TSW. Correlation was calculated. Analyses were performed in Statistic Version 9.0 and R version 3.5.0 (package “corrplot”).

## Results

**FIGURE 2:**
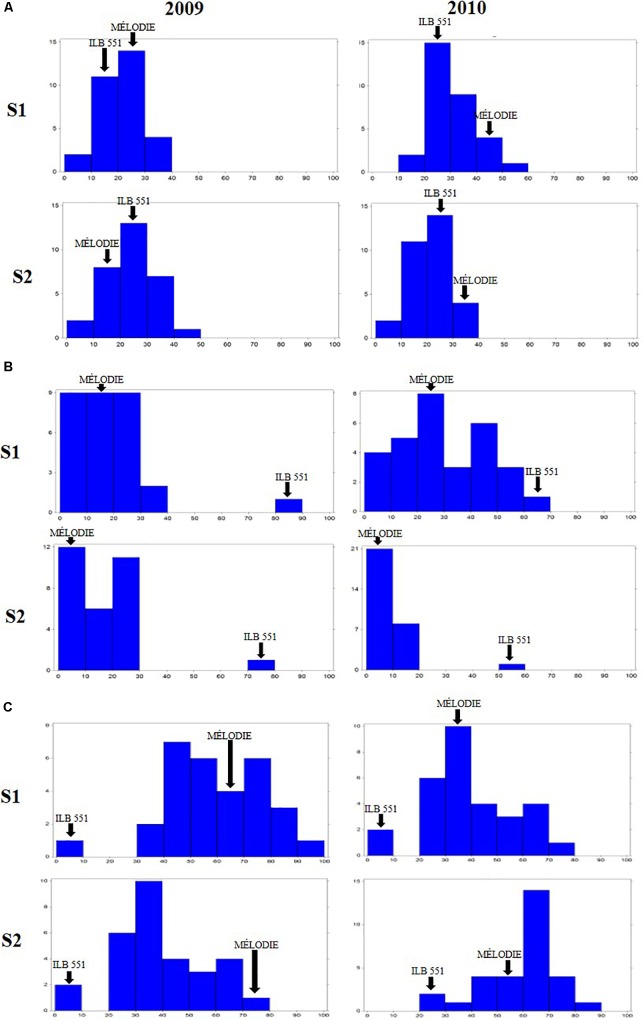
Frequency distribution of the percentage of surface damage **(A)**, emergence holes **(B)**, and healthy seeds **(C)** resulting from *Bruchus* spp. attack in the 29 accessions of faba bean evaluated in the field in Dijon (France) in 2009 and 2010 for two sowing dates for each year. *S1* early sowing. *S2* late sowing. Arrows indicate the means of the moderately susceptible control (MÉLODIE) and the highly susceptible control (ILB 551).

**FIGURE 3:**
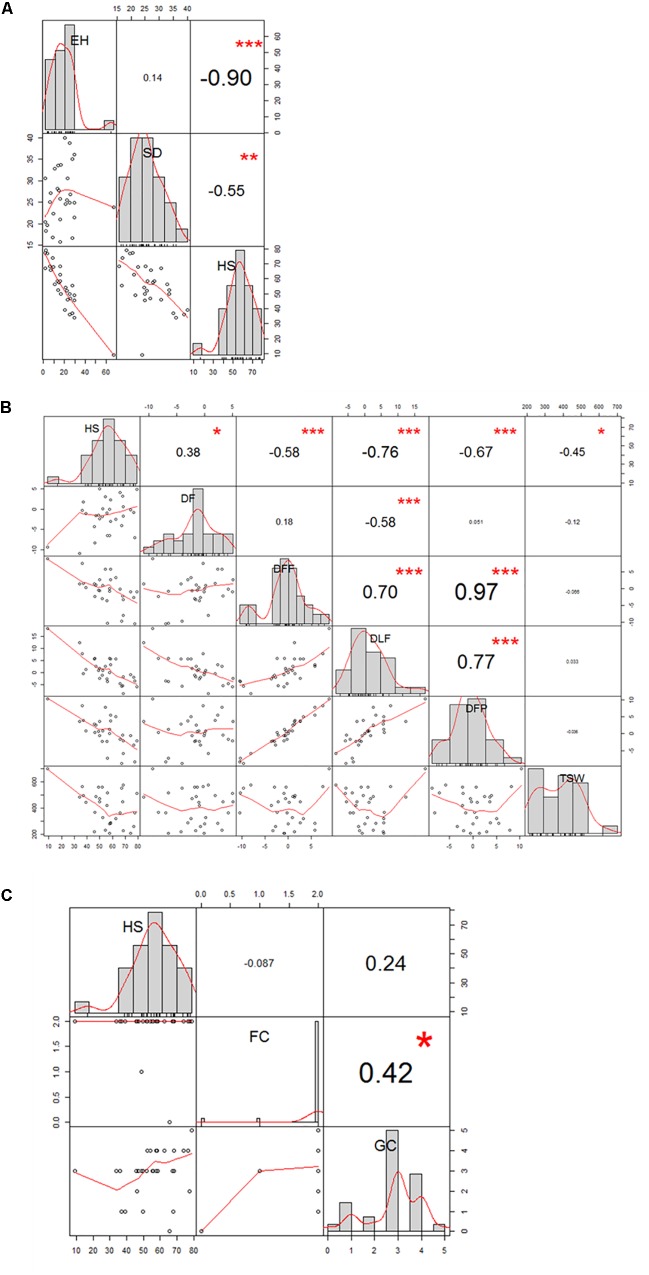
Correlation graph of the traits evaluated in the 29 faba bean accessions studied in the field experiments. **(A)** Spearman correlation between the percentage of healthy seeds (HS), surface damage (SD), and emergence holes (EH); **(B)** Pearson correlation between HS, duration of flowering (DF); days to first flowering (DFF); days to last flowering (DLF); days to first pod-setting(DFP); and thousand-seed weight (TSW); **(C)** Spearman correlation between HS, flower colour (FC), and grain colour (GC).

**FIGURE 4:**
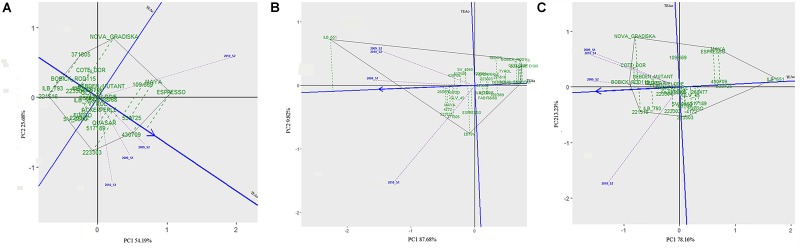
HA-GGE biplots of the percentage of seeds with surface damage **(A)**, emergence holes **(B)**, and healthy seeds **(C)** resulting from *Bruchus* spp. attack in the 29 accessions studied in four environments (combination of year × sowing date). The abscissa axis (Target Environment Coordination abcisa axis, TEAa, blue line) points to the accessions with best performance (shown by a blue arrow, indicating the lowest values for infestation). The ordinates axis or Target Environment Coordination ordinate axis (TEAo; indicated by a horizontal blue line) represents the contribution of each genotype to the G × E interaction. The length of the environmental vectors to the origin (0,0) (indicated by dashed violet lines) will be proportional to the square root of the environmental heritability. The genotype with the best performance would be the one with the lowest/highest values for the evaluated trait (highest negative/positive projection on TEAa) and the most stable throughout all the environments (projection on TEAo close to 0, indicated by green dashed lines); indicating a low G × E interaction. The ideal environment is the one showing the highest projection value onto the TEAa (a long vector indicates more discrimination of principal effects of genotypes) and a small absolute projection value onto the TEAo (projection on TEAo close to 0 indicates more representativeness of all the tested environments in this particular environment).

The 29 accessions evaluated in the field during 2009 and 2010 displayed a quantitative variation of the resistance response to faba bean seed weevil attack (Figure 2 and Supplementary Figure 3). The percentage of surface damage (% SD) ranged from 21.56% in 2009_S2 to 31.84% in 2010_S2 while the percentage of emergence holes (% EH) ranged from 8.84% in the late sowing (S2) 2010 to 29.97% in the early sowing (S1) 2010. The percentage of healthy seeds (% HS) ranged from 40.34% in 2010_S1 to 61.96% in 2009_S2 (Table 2). In 2009, no significant differences (*P* > 0.05) were observed for % SD, % EH, or % HS between the early and late sowing experiments. However, in 2010 the early sowing was related to a higher rate of larvae development (% EH, *P* < 0.05) and less presence of healthy seeds (% HS, *P* < 0.05) compared to the late sowing (Table 2). When comparing early and late sowings of both years (2009 and 2010), it appeared that early sowings were related to a higher percentage of infestation (% EH and % HS, *P* < 0.05) (Table 2). ANOVA revealed highly significant differences between the accessions (G) and the environments (E) (Supplementary Table 2). It also highlighted significant G×E interactions for % SD, % EH, and % HS (Supplementary Table 2). E was the most significant factor affecting the variance of the data, followed by G, and finally by G×E. Broad sense heritability (*H^2^*) was high for % EH (0.93) and % HS (0.82) while, % SD showed a lower heritability (0.56) (Supplementary Table 2). Spearman correlations between % HS-% SD and % HS-% EH were negative (*r* = -0.9 and *r* = -0.55, *P* < 0.001, respectively) (Figure 3A). In contrast, no association between % SD-% EH was observed (*P* > 0.05) (Figure 3A). This was also confirmed by the differences observed between the HA-GGE biplot analyses (Figure 4).

**Table 2:** Mean and standard deviation (SD) of the traits assessed in 29 faba bean accessions in each of the four environments studied.

Trait^a^	Environment^b^	Mean	SD
**SD (%)**	2009_S1	23.07	9.81
	2009_S2	21.56	8.08
	2010_S1	29.69	8.65
	2010_S2	31.84	11.94
	**Average S1**	26.38	9.23
	**Average S2**	26.7	10.01
**EH (%)**	2009_S1	18.19	15.7
	2009_S2	16.48	13.36
	2010_S1	29.97	16.5
	2010_S2	8.84	9.33
	**Average S1**	24.08	16.1
	**Average S2**	12.66	11.345
**HS (%)**	2009_S1	58.75	20.2
	2009_S2	61.96	16.47
	2010_S1	40.34	16.98
	2010_S2	59.32	14.41
	**Average S1**	49.545	18.59
	**Average S2**	60.64	15.44
**TSW (g)**	2009_S1	454.76	150.73
	2009_S2	418.31	143.49
	2010_S1	380.72	143.49
	2010_S2	368.73	139.89
	**Average S1**	417.74	147.11
	**Average S2**	393.52	141.69
**DFF (days)**	2009_S1	–0.83	4.48
	2009_S2	–0.90	5.7
	2010_S1	1.42	5.05
	2010_S2	–1.91	5.02
	**Average S1**	0.30	4.86
	**Average S2**	–1.41	5.35
**DLF (days)**	2009_S1	0.43	6.09
	2009_S2	–0.16	6.50
	2010_S1	2.61	9.06
	2010_S2	1.46	3.25
	**Average S1**	1.52	7.73
	**Average S2**	0.65	5.16
**DF (days)**	2009_S1	–1.26	4.56
	2009_S2	–0.74	4.82
	2010_S1	–1.19	6.80
	2010_S2	–3.38	3.97
	**Average S1**	–1.22	5.74
	**Average S2**	–2.06	4.58
**DFP (days)**	2009_S1	0.15	4.50
	2009_S2	1.03	4.58
	2010_S1	0.45	4.91
	2010_S2	0.49	6.05
	**Average S1**	0.30	4.67
	**Average S2**	0.76	5.32


*^a^SD seeds presenting surface damage, EH Seeds presenting emergence holes, HS healthy seeds, TSW thousand-seed weight, DFF days to first flowering with respect to the control “MÉLODIE,” DLF days to last flowering with respect to the control “MÉLODIE,” DF duration of flowering, DSP days to first pod-setting with respect to the control “MÉLODIE.” ^b^2009_S1 early sowing in 2009, 2009_S2 late sowing in 2009, 2010_S1 early sowing in 2010, 2010_S2 late sowing in 2010.*

### Climatic Factors Most Influencing Faba Bean Seed Weevil Infestation

**FIGURE 5:**
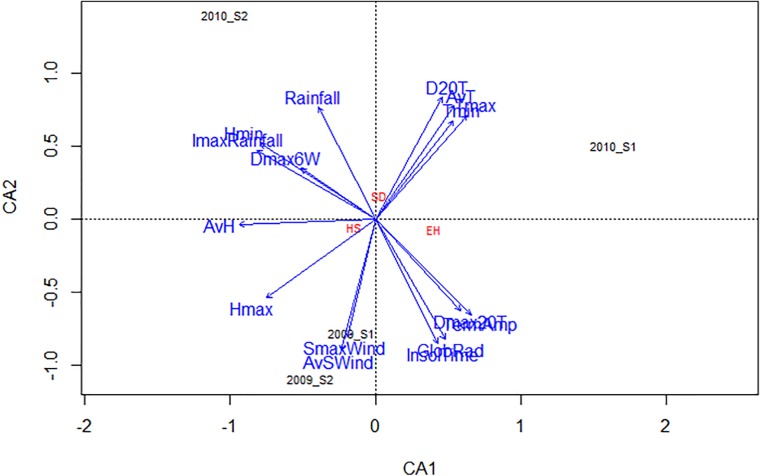
CCA biplot based on the correlation of several climatic parameters with the percentage of surface damage (SD), emergence holes (EH), and healthy seeds (HS) resulting from *Bruchus* spp. attack based on the performance of the 29 faba bean accessions studied in four environments (2009_S1, 2009_S2, 2010_S1, and 2010_S2). *Tmax* maximum temperature, *Tmin* minimum temperature, *AvT* average temperature, *Hmax* maximum relative humidity, *Hmin* minimum relative humidity, *AvH* average relative humidity, *ImaxRainfall* maximum intensity of rainfall, *SmaxWind* maximum speed of wind, *AvSWind* average speed of wind, *D20T* number of days with an average temperature above 20°C, *Dmax20T* number of days in which the maximum temperature has reached or exceeded 20°C, *Dmax6W* number of days when the maximum wind speed has reached or exceeded 6 km/h.

The influence of general weather conditions (Table 1) on % SD, % EH, and % HS for each environment are presented in the CCA biplot (Figure 5). D20T, AvT, Tmin, Tmax, and Rainfall were positively associated with the increase of % SD while, Dmax20T and TermAmp were associated with the decrease of % EH (Figure 5). In addition, Hmax and AvH negatively affected the success of seed weevil attack (decreased % HS) (Figure 5). The climatic factors evaluated had a different influence on the attack of the seed weevils in the environments studied. Thus, whereas SmaxWind and AvSWind were associated with decreased infestation during the early and late sowings of 2009, D20T AvT, Tmin, and Tmax were associated with increased infestation during the early sowing in 2010 and Rainfall was associated with the increased attack during the late sowing of 2010 (Figure 5).

### Phenological and Agronomical Factors Influencing Faba Bean Seed Weevil Infestation

Some phenological and agronomical traits measured on the 29 accessions studied were correlated with faba bean seed weevil infestation. We observed a negatively strong significant linear correlation for % HS-DLF (*r* = -0.76; *P* < 0.001) and % HS-DFP (*r* = -0.67; *P* < 0.001), while a moderately negative significant linear correlation was observed for % HS-DFF (*r* = -0.58; *P* < 0.001) and % HS-TSW (*r* = -0.45; *P* < 0.05) and a weak positive correlation for %HS-DF (*r* = 0.38; *P* < 0.05) (Figure 3B). Moreover, flowering and pod-setting traits resulted to had significantly positive correlations (DFF-DFP, *r* = 0.97, *P* < 0.001; DFF-DLF, *r* = 0.70, *P* < 0.001; and DLF-DFP, *r* = 0.77, *P* < 0.001). A moderately significant negative correlation was observed for DF-DLF (*r* = -0.58; *P* < 0.001). No significant correlation was found between % HS and FC nor between % HS and GC (Figure 3C and Supplementary Figure 4). Nonetheless, significant differences among accessions were found for all these traits (*P* < 0.05).

### Sources of Resistance to Faba Bean Seed Weevil Larvae Penetration

A wide range of values were scored for % SD (Figure 2A and Supplementary Figure 3) for the 29 faba bean accessions evaluated in the different environments, suggesting significant seed weevil susceptibility differences among the accessions (Supplementary Table 2). The accessions “221516” (15.56%), “ILB 793” (18.78%), “4172” (20.11%) and “BOBICK ROD115” (21%) showed the best and most stable performance in the different environments (Supplementary Figure 3). By contrast, the accessions “ESPRESSO” (41.62%), “430709” (38.22%), “109.669” (37%), and “533725” (37%) were the most susceptible accessions in the environments studied (Supplementary Figure 3). This was confirmed by the classification resulting from the HA-GGE biplot analysis (Figure 4A), where the first two principal components explained 79.53% of the variance of the data. The HA-GGE biplot proved adequate to explain the interactions of G×E according to Yang et al. (2009) because (G+GE)/(E+G+GE) > 10% (Supplementary Table 2). The accessions with the highest negative projection on the TEAa (abcisa axis of the target environment coordination) showed the lowest values for % SD and those with a projection on TEAo (ordinate axis of the target environment coordination) closest to the origin were the most stable throughout all the environments, indicating a low G×E interaction (Figure 4A). The furthest accessions from the biplot origin delimited the vertices of a polygon (black line Figure 4A) including accessions displaying the greatest responses to environmental changes (less stable). These were the accessions “ESPRESSO,” “430709,” and “22303” (21.78%) among the most susceptible and “NOVA GRADISKA” (25.89%), “371805” (17.67%), and “221516” 677 (15.56%) among the most resistant. Table 3 presents the 10 accessions with the best performance for % SD (the lowest % SD and most stable) based on the HA-GGE analysis (Figure 4A). This agreed with the classification resulting from the average % SD values for the four environments with slight variations in the ranking positions (Supplementary Figure 3).

**Table 3:** Ranking of the 10 faba bean accessions with the lowest percentages of surface damage (SD) and emergence holes (EH) and the highest percentage of healthy seeds (HS) after *Bruchus* spp. infestation, based in the HA-GGE biplot analysis taking into account their performance and stability among the four environments studied.

		SD (%)	EH (%)	HS (%)
	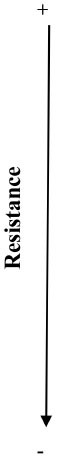	ILB 793	CÔTE D’OR	BOBICK ROD115
		221516	QUASAR	CÔTE D’OR
		BOBICK ROD115	NOVA GRADISKA	NOVA GRADISKA
		371805	BOBICK ROD115	221516
		NOVA GRADISKA	DEBDEN MUTANT	ILB 793
		CÔTE D’OR	TYROL	DEBDEN MUTANT
		DEBDEN MUTANT	221516	REDOS
		223304	109.669	QUASAR
		REDOS	REDOS	TYROL
		GLV 45	223303	223304


### Sources of Resistance to Faba Bean Seed Weevil Larvae Development

The percentage of seeds with emergence holes (% EH) revealed the accessions “BOBICK ROD 115” (2.22%), “CÔTE D’OR” (2.33%), “QUASAR” (2.8%) and “NOVA GRADISKA” (4.67%) as the best and most stable across the environments (Supplementary Figure 3). On the contrary, “ILB551” (61.26%), “533725” (31.11%) “MAYA” (29.97%) and “268477” (29.22%) were the most susceptible accessions (Supplementary Figure 3). This agreed with the results of the HA-GGE biplot analysis (Figure 4B) that explained 97.57% of the variance in the two first principal components (PC1 and PC2) with (G+GE)/(E+G+GE) > 10% (Supplementary Table 2). The ten best accessions (Table 3) resulted from the HA-GGE analysis (Figure 4B) also agreed with the classification derived from average values for the 4 environments with slight variations in the ranking positions (Supplementary Figure 3). Among these accessions, those highly affected by the environmental conditions were “ILB 551”and “EBT0V” among the susceptible and “BOBICK ROD115” and “CÔTE D’OR” among the resistant accessions (Figure 4B).

### Sources of Resistance to Faba Bean Seed Weevil Infestation

The percentage of healthy seeds (% HS) was related to the two previous variables (% HS = 100 - % EH-% SD). A wide range of values was scored for % HS among the 29 faba bean accessions evaluated in the four environments (Supplementary Figure 3). “BOBICK ROD115” (76.78%), “221516” (74.22%), “CÔTE D’OR” (73.67%), and “NOVA GRADISKA” (69.44%) were the best and most stable accessions across the environments (Supplementary Figure 3). This was confirmed by the HA-GGE biplot analysis (Figure 4C) where the PC1 and PC2 accounted for 91.38% of the variance (Figure 4C) with (G+GE)/ (E+G+GE) > 10% (Supplementary Table 2). “NOVA GRADISKA” and “221516” showed the greatest variance among the accessions with the highest % HS; while “MAYA” (33.24%) and “223303” (52.33%) presented the greatest variance among the accessions with the lowest % HS (Figure 4C). The 10 best accessions resulting from the HA-GGE biplot analysis (Figure 4C) are presented in Table 3. Slight variations in the ranking positions were observed between the biplot analysis (Figure 4C) and the average values for the four environments (Supplementary Figure 3).

## Discussion

The phenotyping of germplasm collections is crucial for the progress of sustainable agriculture since they constitute the reservoir in which new sources of resistance may be identified. Bruchid resistance is of special relevance to faba bean world production due to the important losses caused by seed weevils. The identification and introgression of resistance genes into cultivars appears to be the best option to control bruchids in the field. Therefore, the objective of this work was to identify sources of resistance to faba bean seed weevil attacks in a *V. faba* germplasm collection available at INRA, France. After two series of phenotyping under natural conditions, the most resistant accessions were evaluated in two field experiments conducted in two consecutive years. Each year, an early and a late sowing date allowed determining the effect of the sowing date on the infestation rate and the climatic factors that most influenced the incidence of the attack. The infested seeds presented two types of damage: (1) seeds in which the larvae perforated the seed coat but did not develop inside the cotyledons (percentage of surface damage, % SD) (Figure 1B) and (2) seeds where larvae reached the imago (adult) stage (percentage of emergence holes, % EH) (Figures 1C–E).

The screening of this large *V. faba* germplasm collection successfully identified useful sources of resistance to faba bean seed weevils. In addition, our study showed the impact of the environment on the rate of infestation and reported on climatic factors as well as the phenological and agronomic traits that most influenced the incidence of weevil attack.

### Temperature, Humidity, and Rainfall Are the Most Influential Climatic Factors in Infestation by Faba Bean Seed Weevils

Temperature-related variables, rainfall, and maximum humidity (Figure 5) were the most influential climatic factors affecting seed infestation. Rainfall and humidity potentially disturb oviposition and detach the eggs from the surface of the pods, while high temperatures desiccate the eggs thus reducing their viability (Roubinet, 2016; Aznar-Fernández et al., 2017). Taking into account these climatic parameters when choosing the location and timing of field experiments could be useful for breeders and researchers when selecting for resistance to bruchids.

### Early Sowings Showed the Highest Rates of Infestation by Faba Bean Seed Weevils

Early sowings were associated with higher rates of infestation than late sowings (Table 2). However, the proximity between early sowing and late sowing plots could have influenced the infestation rate in the late sowing experiment. Therefore, new experiments where early sown and late-swon plots would be geographically distant should be carried out to avoid any effect of early sowing plants on the infestation of the late sowing plants. Previous works on *Bruchus* sp. have shown controversial results regarding the effect of sowing date on the incidence of the attacks. The effect of sowing date ranged from null (Newman and Elliot, 1938; Gerding et al., 1987) to a lower rate of infestation in early sowings (Tahhan and Van Emden, 1989) or a lower rate of infestation in late sowings (Brindley, 1933; Newman et al., 2011; Szafirowska, 2012). This could be due to the fact that the effect of the sowing date on the infestation depends on the climatic characteristics of the trial, as our work shows.

### Early Flowering, Early Pod-Setting and Low Thousand-Seed Weight Accessions Are Correlated With Greater Resistance to Faba Bean Seed Weevils

Faba bean flowers are key to female insect sexual maturation and pods are the substrate on which females oviposit their eggs (Medjdoub-Bensaad et al., 2007; Leppik et al., 2014; Roubinet, 2016). The timing of flowering and pod-setting had a crucial impact on seed weevil infestation. The accessions that bloomed earlier than “MÉLODIE” also developed pods earlier while late flowering accessions developed pods later: 94% of the variation in % DFP was related to the variation in DFF (Figure 3B). Earlier accessions compared to the semi-early and moderately susceptible control “MÉLODIE” were associated with lower seed weevil infestation. 33.6, 57.8, and 44.9% of the variation of % HS could be explained by DFF, DLF, and DFP, respectively. “DEBDEN MUTANT” was the only accession to flower significantly earlier than “MÉLODIE” and together with “QUASAR,” “106.669,” and “CÔTE D’OR” presented significantly lower DFP than the control. However, no significant differences (*P* > 0.05) were found for % HS between these early accessions and “MÉLODIE.” Thus, an escape based in an asynchrony between flowering and/or pod-setting (Aznar-Fernández et al., 2017) seemed to not be involved in the response of these accessions to seed weevil infestation. The most likely explanation to this general tendency is that the climatic conditions at the flowering time of the early accessions were not the most appropriate for the insect to interrupt the diapause (Medjdoub-Bensaad et al., 2007). Temperature appeared to be the most differentiating climatic factor between the early and late accessions, observing that early accessions bloomed and developed pods at colder temperatures than late accessions. Late accessions needed warmer conditions, which are more optimal for the biology of faba bean seed weevils. The accessions with long periods of flowering (DF) resulted to be also early accessions (DLF) with respect to“MÉLODIE,” so 33.6% of the variation in DF could be explained by their variation in DLF. The time during which flowers were available for seed weevils to feed showed a weak correlation with the level of infestation (% HS) (Figure 3B), observing that 14.4 % of the variation of % HS may be related to the variation in DF. Interestingly, the late accessions “ILB 551” (highly susceptible control) and “533725” were the most susceptible besides presenting a restricted time of flowering. It would be interesting to deepen the study of these accessions to reveal the factors involved in their preference by bruchids because it could facilitate the development of new tools that limit infestations as the previously developed by Frérot et al. (2017) (Leppik et al., 2014) based on semiochemical attractants that trap the insects (Leppik et al., 2014). No significant differences for DF were observed among the rest of the accessions studied, although % HS ranged from 34% to 79% to in these accessions.

The thousand-seed weight (TSW) is one of the most important traits for faba bean breeding due to its correlation with crop yield (Toker, 2004). However, breeding for yield has been shown to lead to an indirect loss of insect resistance by reducing defense barriers and/or favoring the development of insects (Holt and Birch, 1984). Faba bean germplasm accessions have a smaller size and weight than elite cultivars (Cubero, 1974; Duc, 1997). In our study, the accessions with lower TSW presented high % HS within a moderate correlation: the variation of TSW explained 20.2% of % HS variation. Nonetheless, the accessions “BOBICK ROD115,” “DEBDEN MUTANT,” “REDOS,” or “QUASAR” presented a significantly higher TSW than that of “MÉLODIE” besides having a low percentage of infestation which makes them interesting as progenitors for breeding selection.

### Identification of Novel Sources of Resistance to Faba Bean Seed Weevils

Of all the accessions that were examined in our study, four accessions exhibited low infestation levels (low % EH and SD) and good stability among the environments assessed (combination of year and sowing date): the inbred line “BOBICK ROD115” (*V. faba* var. equina) and the traditional landraces “CÔTE D’OR” (*V. faba* var. minor), “221516” (*V. faba* var. major) and “NOVA GRADISKA” (*V. faba* var. minor). Other interesting sources of resistance against seed weevil attacks were the accessions “QUASAR” (*V. faba* var. equina), “109.669” (*V. faba* var. equina), and “223303” (*V. faba* var. minor) that recorded a low success of metamorphosis of the larvae into imago despite presenting considerable surface damage (% SD) (Figure 4 and Supplementary Figure 3). The wide range of infestation levels observed among the best resistant accessions suggests the involvement of different defense mechanisms (Figure 4). Antibiosis and/or antixenosis mechanisms may have been acting to prevent, retard and/or hinder oviposition, penetration of the pod and seed coat or the development of the larvae (Lattanzio et al., 2005). These include physical or mechanical barriers such as the thickness, hardness, or texture of the pod that can hinder the adherence of the eggs and limit access into the seed (Lephale et al., 2012). The seed coat can also hamper penetration into the seed because it contains biochemical defense barriers (alkaloids, polyphenols, lectins, proteinase inhibitors, α-amylase inhibitors, etc.) involved in the reduction of fertility and/or oviposition, the increase of development time and/or mortality of larvae or adults (Mishra et al., 2018). The accessions “QUASAR,” “109.669,” and “223303” that showed a low % EH but high % SD, could trigger very early and/or intense antibiosis mechanisms that prevent the larvae that have managed to penetrate the seed coat from developing inside (Macel and Dam, 2018). The case of “QUASAR” is interesting because the success rate of larval metamorphosis into adults was very low (% SD >> % EH). In particular, the agronomic potential of this accession presenting the highest seed weight among the resistant accessions, is great for breeding (Casler and Vogel, 1999; Chen et al., 2015). In addition to defense mechanisms, escape through precocity (as stated above) has been described as a possible cause of % HS variation (Aznar-Fernández et al., 2017). In our study, the most resistant accessions bloomed at intermediate dates with respect to the rest of the evaluated accessions (*P* > 0.05) and there were no significant differences (*P* > 0.05) for DFF among them, which suggest that the escape was not related to their response to the attack.

The introgression of seed weevil resistance genes in faba bean cultivars is a major challenge, because faba bean trade does not admit damage to the grain. Identifying different sources of resistance triggering different types of defense mechanisms to be introgressed simultaneously in cultivars will make the resistance more durable and suitable for sustainable agriculture with limited use of pesticides. The present work has identified different sources of resistance that could be used as progenitors in faba bean breeding programs. No genotype showed complete resistance, so pyramiding resistance genes is important. The next steps will be aimed at determining how resistance is inherited and what specific defense mechanisms are acting. “BOBICK ROD115,” “NOVA GRADISKA,” “CÔTE D’OR,” or “QUASAR” will be very useful toward this aim because their different response to the attack suggests a distinct basis of seed weevil resistance. The wide range of resistance levels observed among the accessions studied and the fact that complete resistance has not been identified suggest a complex inheritance of the trait. This will be confirmed through genetic analyses that will be performed in two different recombinant inbred line populations (RILs) that have respectively “NOVA GRADISKA” and “QUASAR” as one of the parents. These analyses will also reveal potential candidate genes for resistance to faba bean weevils.

## Author Contributions

EC-P analyzed the data and wrote the manuscript. BR, DO, CD, and J-BM-R conducted the experiments. NT reviewed the manuscript. PM conceived, supervised, and reviewed research and the manuscript. All authors read and approved the manuscript.

## Conflict of Interest Statement

The authors declare that the research was conducted in the absence of any commercial or financial relationships that could be construed as a potential conflict of interest.

## References

[B1] Adam J. I. Baidoo P. K. (2008). Susceptibility of five cowpea (*Vigna unguiculata*) varieties to attack by callosobruchus maculatus (Fab.) [Coleoptera: Bruchidae]. *J. Ghana Sci. Assoc.* 10 85–92.

[B2] Amzouar S. Boughdad A. Maatoui A. Allam L. (2016). Comparison of the chemical composition and the insecticidal activity of essential oils of *Mentha suaveolens* Ehrh. collected from two different regions of Morocco, against *Bruchus rufimanus* (Bohman) (Coleoptera: Chrysomelidae). *Int. J. Innov. Appl. Sci.* 18 836–845.

[B3] Athiepacheco I. Bolonhezi S. Sartori M. R. Turatti de Paula D. C. Lourenção A. L. (1994). Resistência a bruquídeos, composição em ácidos graxos e qualidade de cozimento das sementes em genótipos de grão-de-bico. *Bragantia* 53 61–74. 10.1590/S0006-87051994000100007

[B4] Aznar-Fernández T. Carrillo-Perdomo E. Flores F. Rubiales D. (2017). Identification and multi-environment validation of resistance to pea weevil (*Bruchus pisorum*) in *Pisum germplasm*. *J. Pest Sci.* 91 505–514. 10.1007/s10340-017-0925-1

[B5] Brindley T. A. (1933). Some notes on the biology of the pea weevil *Bruchus pisorum* L. (Coleoptera, Bruchidae) at Moscow, Idaho. *J. Econ. Entomol.* 26 1058–1062. 10.1093/jee/26.6.1058

[B6] Cabrera A. Martin A. (1986). Variation in tannin content in *Vicia faba* L. *J. Agric. Sci.* 106 377–382. 10.1017/S0021859600063978 21391607

[B7] Casler M. D. Vogel K. P. (1999). Accomplishments and impact from breeding for increased forage nutritional value. *Crop Sci.* 39 12–20. 10.2135/cropsci1999.0011183X003900010003x

[B8] Chen Y. H. Gols R. Benrey B. (2015). Crop domestication and its impact on naturally selected trophic interactions. *Annu. Rev. Entomol.* 60 35–58. 10.1146/annurev-ento-010814-020601 25341108

[B9] Christensen C. M. Kaufmann H. H. (1965). Deterioration of stored grains by fungi. *Annu. Rev. Phytopathol.* 3 69–84. 10.1146/annurev.py.03.090165.000441

[B10] Clement S. L. Hardie D. Elberson L. R. (2002). Variation among accessions of for resistance to pea weevil. *Crop Sci.* 42 2167–2173. 10.2135/cropsci2002.2167

[B11] Cubero J. I. (1974). On the evolution of *Vicia faba* L. *Theor. Appl. Gen.* 45 47–51. 10.1007/BF00283475 24419274

[B12] Dongre T. K. Pawar S. E. Thakare R. G. Harwalkar M. R. (1996). Identification of resistant sources to cowpea weevil (*Callosobruchus maculatus* (F.)) in *Vigna* sp. and inheritance of their resistance in black gram (*Vigna mungo* var. mungo). *J. Stored Prod. Res.* 32 201–204. 10.1016/S0022-474X(96)00028-8

[B13] Duc G. (1997). Faba bean(*Vicia faba* L.). *Field Crops Res.* 53 99–109. 10.1016/S0378-4290(97)00025-7

[B14] Duc G. Bao S. Baum M. Redden B. Sadiki M. Suso M. J. (2010). Diversity maintenance and use of *Vicia faba* L. genetic resources. *Field Crops Res.* 115 270–278. 10.1016/J.FCR.2008.10.003

[B15] Frérot B. Leppik E. Groot A. T. Unbehend M. Holopainen J. K. (2017). Chemical signatures in plant–insect interactions. *Adv. Bot. Res.* 81 139–177. 10.1016/BS.ABR.2016.10.003

[B16] Gerding M. Tay J. Paredes M. (1987). *Incidence of Bruchus pisorum L. (Coleoptera: Bruchidae) on Pea, According to Seeding Date and Density*. Santiago: Instituto de Investigaciones Agropecuarias.

[B17] Goossens A. Quintero C. Dillen W. De Rycke R. Flower Valor J. De Clercq J. (2000). Analysis of bruchid resistance in the wild common bean accession G02771: no evidence for insecticidal activity of arcelin 5. *J. Exp. Bot.* 51 1229–1236. 10.1093/jexbot/51.348.1229 10937698

[B18] Hoffman A. Labeyrie V. Balachowsky A. S. (1962). “Famille des bruchidae,” in *Entomologie Appl. À l’agriculture*, ed. Balachowsky A. S. (Paris: Masson et Cie), 434–494.

[B19] Holt J. Birch N. (1984). Taxonomy, evolution and domestication of Vicia in relation to aphid resistance. *Ann. Appl. Biol.* 105 547–556. 10.1111/j.1744-7348.1984.tb03081.x

[B20] Hulme P. E. (2009). “Handbook of alien species in Europe, invading nature,” in *Springer Series in Invasion Ecology*, ed. Drake J. A. (Dordrecht:Springer).

[B21] Ishimoto M. Chrispeels M. J. (1996). Protective mechanism of the Mexican bean weevil against high levels of alpha-amylase inhibitor in the common bean. *Plant Physiol.* 111 393–401. 10.1104/PP.111.2.393 8787024 PMC157848

[B22] Jadhav D. R. Mallikarjuna N. Rao G. V. R. (2012). *Callosobruchus maculatus* resistance in some wild relatives and interspecific derivatives of Pigeonpea. *Indian J. Plant Prot.* 40 40–44.

[B23] Jemâa J. M. B. (2014). Essential oil as a source of bioactive constituents for the control of insect pests of economic importance in tunisia screening of factors influencing the efficacy of *Pistacia lentiscus* (L.) essential oil from Tunisia for the control of *Tribolium castaneu*. *Med. Aromat. Plants* 3:158 10.4172/2167-0412.1000158

[B24] Keneni G. Bekele E. Getu E. Imtiaz M. Damte T. Mulatu B. (2011). Breeding food legumes for resistance to storage insect pests: potential and limitations. *Sustainability* 3 1399–1415. 10.3390/su3091399

[B25] Kergoat G. J. Silvain J.-F. Delobel A. Tuda M. Anton K.-W. (2007). Defining the limits of taxonomic conservatism in host–plant use for phytophagous insects: molecular systematics and evolution of host–plant associations in the seed-beetle genus *Bruchus Linnaeus* (Coleoptera: Chrysomelidae: Bruchinae). *Mol. Phylogenet. Evol.* 43 251–269. 10.1016/J.YMPEV.2006.11.026 17276089

[B26] Kharrat M. Le Guen J. Tivoli B. (2006). Genetics of resistance to 3 isolates of *Ascochyta fabae* on Faba bean (*Vicia faba* L.) in controlled conditions. *Euphytica* 151 49–61. 10.1007/s10681-006-9127-2

[B27] Lattanzio V. Terzano R. Cicco N. Cardinali A. Di Venere D. Linsalata V. (2005). Seed coat tannins and bruchid resistance in stored cowpea seeds. *J. Sci. Food Agric.* 85 839–846. 10.1002/jsfa.2024

[B28] Lephale S. Addo-Bediako A. Ayodele V. (2012). Susceptibility of seven cowpea cultivars (*Vigna unguiculata*) to cowpea beetle (*Callosobruchus maculates*). *Agric. Sci. Res. J.* 2 65–69.

[B29] Leppik E. Pinier C. Frerot B. (2014). “Chemical landscape of agro-biocoenosis: case study of broad faba bean and its specialized pest *Bruchus rufimanus*,” in *Proceedings of the 10th International Conference on Agricultual Pests*, Montpellier.

[B30] Maalouf F. Hamdi A. Cubero J. I. Khalifa G. E. Jarsso M. Kemal S. (2009). *Development of Faba Bean Productivity and Production in the Nile Valley, Red Sea And Sub-Saharan Region*. Aleppo: ICARDA.

[B31] Macel M. Dam N. M. V. (2018). “Metabolomics of plant resistance to insects,” in *The Biology of PLant-Insect Interactions: A Compendium for the Plant Biotechnologist*, ed. Emani C. (Boca Raton, FL: Science Publishers - CRC Press), 129–149. 10.1201/9781315119571-7

[B32] McIntosh M. S. (1983). Analysis of combined experiments. *Agron. J.* 75 153–155. 10.2134/agronj1983.00021962007500010041x

[B33] Medjdoub-Bensaad F. Frah N. Huignard J. (2015). Dynamique des populations de la bruche de la fève, *Bruchus rufimanus* (Coleoptera: Chrysomelidae), durant la période d ’ activité reproductrice et de diapause. *Nat. Technol.* 13 12–21.

[B34] Medjdoub-Bensaad F. Khelil M. A. Huignard J. (2007). Bioecology of broad bean bruchid *Bruchus rufimanu*s Boh. (Coleoptera: Bruchidae) in a region of Kabylia in Algeria. *Afr. J. Agric. Res.* 2 412–417.

[B35] Mishra S. K. Macedo M. L. R. Panda S. K. Panigrahi J. (2018). Bruchid pest management in pulses: past practices, present status and use of modern breeding tools for development of resistant varieties. *Ann. Appl. Biol.* 172 4–19. 10.1111/aab.12401

[B36] Morton R. L. Schroeder H. E. Bateman K. S. Chrispeels M. J. Armstrong E. Higgins T. J. V. (2000). Bean a-amylase inhibitor 1 in transgenic peas (*Pisum sativum*) provides complete protection from pea weevil (*Bruchus pisorum*) under field conditions. *Proc. Natl. Acad. Sci. U.S.A.* 97 3820–3825. 10.1073/pnas.070054597 10759552 PMC18100

[B37] Mulualem T. Dessalegn T. Dessalegn Y. (2012). Participatory varietal selection of faba bean (*Vicia faba* L.) for yield and yield components in Dabat district. *Ethiopia. Wudpecker J. Agric. Res.* 7 270–274.

[B38] Newman L. J. Elliot H. G. (1938). The pea weevil, *Bruchus pisorum* (Linn.). *J. Dept. Agr. Western Austr.* 15 156–158.

[B39] Newman S. M. Tantasawat P. Steffens J. C. (2011). Tomato polyphenol oxidase B is spatially and temporally regulated during development and in response to ethylene. *Molecules* 16 493–517. 10.3390/molecules16010493 21224781 PMC6259212

[B40] Redden R. Dobie P. Gatehouse A. (1983). The inheritance of seed resistance to *Callosobruchus maculatus* F. in cowpea (*Vigna unguiculata* L. Walp.). I. Analyses of parental, F 1, F 2, F 3 and backcross seed generations. *Aust. J. Agric. Res.* 34 681–696. 10.1071/AR9830681

[B41] Roubinet E. (2016). *Management of the Broad Bean Weevil (Bruchus rufimanus Boh.) in Faba Bean (Vicia faba L.)*. Uppsala: Sveriges lantbruksuniversitet Swedish University of Agricultural Sciences.

[B42] Sallam M. N. (2013). *INSECT DAMAGE: Damage on Post-Harvest*, eds Mejia D. Lewis B. (Rome: Food and Agriculture Organization of the UnitedNations).

[B43] Schafleitner R. Huang S. Chu S. Yen J. Lin C. Yan M. (2016). Identification of single nucleotide polymorphism markers associated with resistance to bruchids (*Callosobruchus* spp.) in wild mungbean (*Vigna radiata* var. sublobata) and cultivated V. radiata through genotyping by sequencing and quantitative trait locus analysis. *BMC Plant Biol.* 16:159. 10.1186/s12870-016-0847-8 27422285 PMC4946214

[B44] Seidenglanz M. Huňady I. (2016). Effects of faba bean (*Vicia faba*) varieties on the development of *Bruchus rufimanus*. *Czech J. Genet. Plant Breed.* 52 22–29. 10.17221/122/2015-CJGPB

[B45] Seidenglanz M. Huňady I. Sillero J. C. (2017). “Testing of *Vicia faba* accessions on resistance to bruchids (*Bruchus rufimanus*),” in *Proceedings of the International Conference Advances in Grain Legume Breeding, Cultivations and Uses for a More Competitive Value-Chain*, Novi Sad.

[B46] Shaheen F. A. Khaliq A. Aslam M. (2006). Resistance of chickpea (*cicer arietinum* l.) cultivars against pulse beetle. *Pak. J. Bot.* 38 1237–1244.

[B47] Szafirowska A. (2012). The role of cultivars and sowing date in control of broad bean weevil (*Bruchus Rufimanus* Boh.) in organic cultivation. *Veg. Crops Res. Bull.* 77 29–36. 10.2478/v10032-012-0013-2

[B48] Tahhan O. Van Emden H. F. (1989). Resistance of faba bean, *Vicia faba*, to *Bruchus dentipes* Baudi (Coleoptera: Bruchidae). *Bull. Entomol. Res.* 79 211–218. 10.1017/S0007485300018198

[B49] Titouhi F. Amri M. Messaoud C. Haouel S. Youssfi S. Cherif A. (2017). Protective effects of three *Artemisia* essential oils against *Callosobruchus* maculatus and *Bruchus rufimanus* (Coleoptera: Chrysomelidae) and the extended side-effects on their natural enemies. *J. Stored Prod. Res.* 72 11–20. 10.1016/J.JSPR.2017.02.007

[B50] Toker C. (2004). Estimates of broad-sense heritability for seed yield and yield criteria in faba bean ( *Vicia faba* L.). *Hereditas* 225 222–225. 10.1111/j.1601-5223.2004.01780.x 15198712

[B51] Tran B. Darquenne J. Huignard J. (1993). Changes in responsiveness to factors inducing diapause termination in *Bruchus* rufimanus (Boh.) (Coleoptera: Bruchidae). *J. Insect Physiol.* 39 769–774. 10.1016/0022-1910(93)90052-S

[B52] Tran B. Huignard J. (1992). Interactions between photoperiod and food affect the termination of reproductive diapause in *Bruchus rufimanus* (Boh.), (Coleoptera, Bruchidae). *J. Insect Physiol.* 637 639–642. 10.1016/0022-1910(92)90115-T

[B53] Yan W. Holland J. B. (2010). A heritability-adjusted GGE biplot for test environment evaluation. *Euphytica* 171 355–369. 10.1007/s10681-009-0030-5 26489689

[B54] Yan W. Rajcan I. (2002). Biplot analysis of test sites and triat relations of soybean in Ontario. *Crop Sci.* 42 11–20. 10.2135/cropsci2002.1100 11756248

[B55] Yang R. C. Crossa J. Cornelius P. L. Burgue J. (2009). Biplot analysis of genotype × environment interaction: Proceed with caution. *Crop Sci.* 49 1564–1576.

