# Supplementary material for: Identification of Novel Sources of Resistance to Seed Weevils (Bruchus spp.) in a Faba Bean Germplasm Collection
Source: Front Plant Sci. 2019 Jan 9;9:1914. doi: 10.3389/fpls.2018.01914 (PMC6333698; doi:10.3389/fpls.2018.01914)
Supplement: Supplementary file 2 [file Table_2.pdf]

Table 2. Accession (G), sowing date-year (E) and GxE interaction of the percentage of seeds with surface damage (SD) and emergence holes (EH) and the percentage of healthy seeds (HS) in the faba bean field trials for the assessment of *Bruchus* spp. infestation.

**Trait**	**Source**	**df**	**Mean squares**	**F**	***H***	**G+GE//(E+G+GE)**
**SD (%)**	(E	3	22.97***	34.15	0.56	0.65
	Block	5	0.95			
	G	29	5.51***	8.19		
	G x E	87	2.22**	3.30		
	Error	238	0.67			
	Total	359				
**EH (%)**	E	3	99.62***	196.43	0.93	0.7
	Block	2	4.03			
	G	29	27.53***	54.29		
	G x E	87	1.39***	2.75		
	Error	238	0.51			
	Total	359				
**HS (%)**	E	3	46.94***	99.66	0.82	0.67
	Block	5	0.06			
	G	29	19.60***	41.63		
	G x E	87	1.78***	3.78		
	Error	238	0.47			
	Total	359				

*Significant at the 0.05 level of probability.
**Significant at the 0.01 level of probability.
***Significant at the 0.001 level of probability.
