# Supplementary material for: Identification of Novel Sources of Resistance to Seed Weevils (Bruchus spp.) in a Faba Bean Germplasm Collection
Source: Front Plant Sci. 2019 Jan 9;9:1914. doi: 10.3389/fpls.2018.01914 (PMC6333698; doi:10.3389/fpls.2018.01914)
Supplement: Supplementary file 3 [file Image_1.pdf]

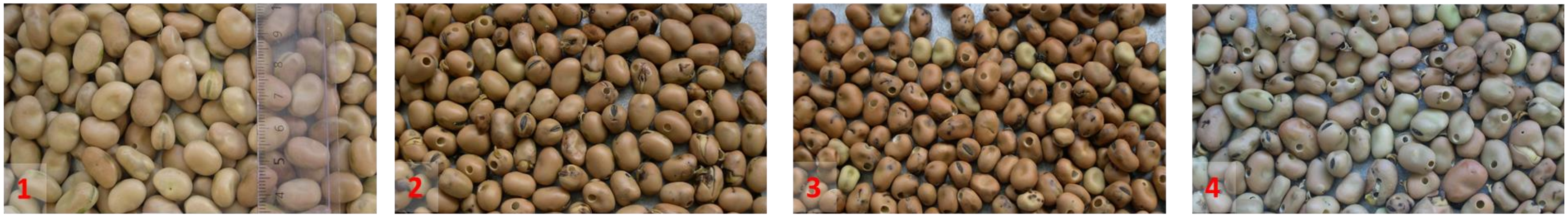

Figure 1 Classification of the faba bean accessions studied into 4 scoring groups based on the levels of *Bruchus* spp. infestation. 0-25 % of infested seeds = 1; 26-50 % of infested seeds= 2; 51-75% of infested seeds= 3; 76-100% of infested seeds= 4.

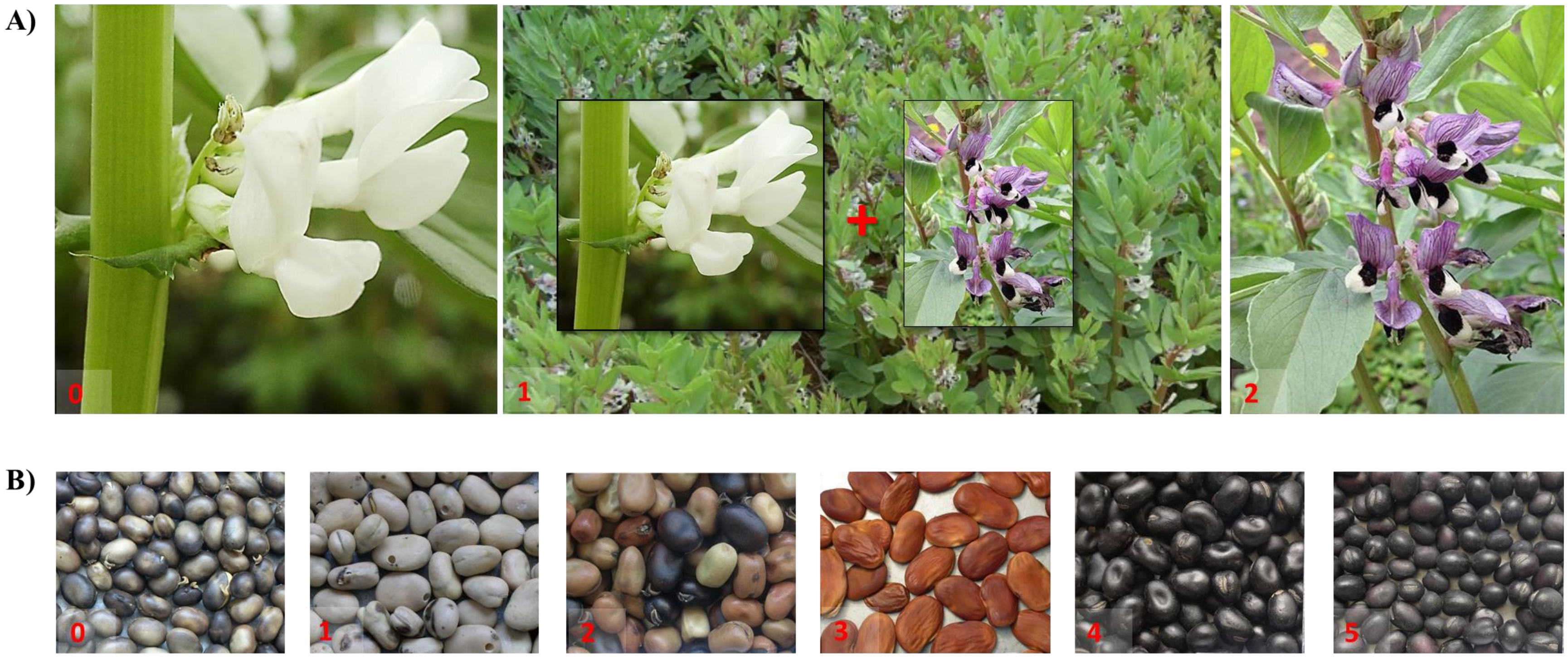

Figure 2. Scales used to evaluate the colour of A) the flowers and B) the seeds of the twenty nine faba bean accessions assessed in the four environments studied. A) White flower = 0; colour segregation = 1 and coloured flower = 2. B) Grey = 0, beige = 1, colour segregation = 2, brown = 3, dark-brown = 4 and dark-violet = 5.

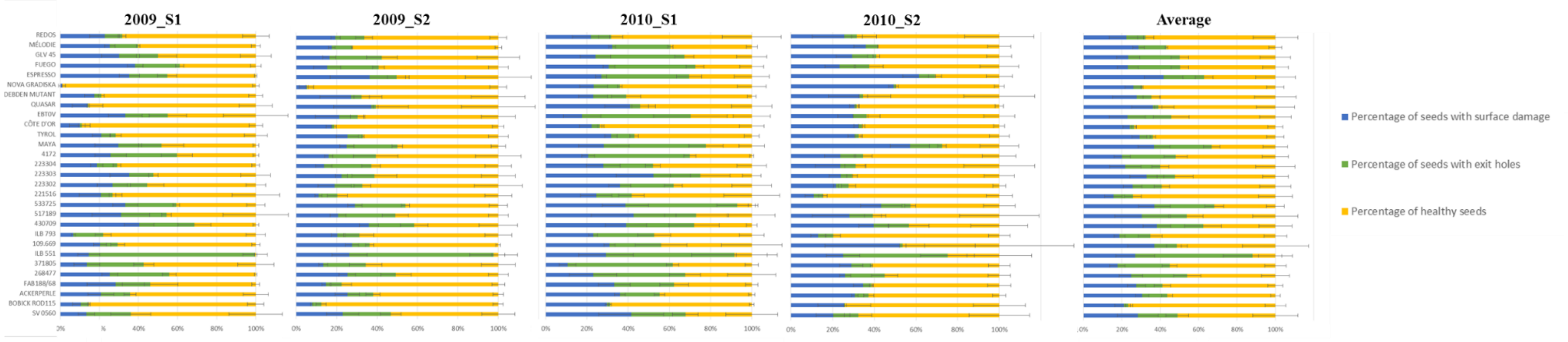

Figure 3. Mean and standard deviation (SD) of the percentages of seeds with surface damage *(blue*), with emergence holes (*green*) and healthy seeds (*yellow*) due to *Bruchus* spp. infestation in 29 faba bean accessions evaluated under field conditions in 4 different environments (2009_S1, 2009_S2, 2010_S1 and 2010_S2) and its average.

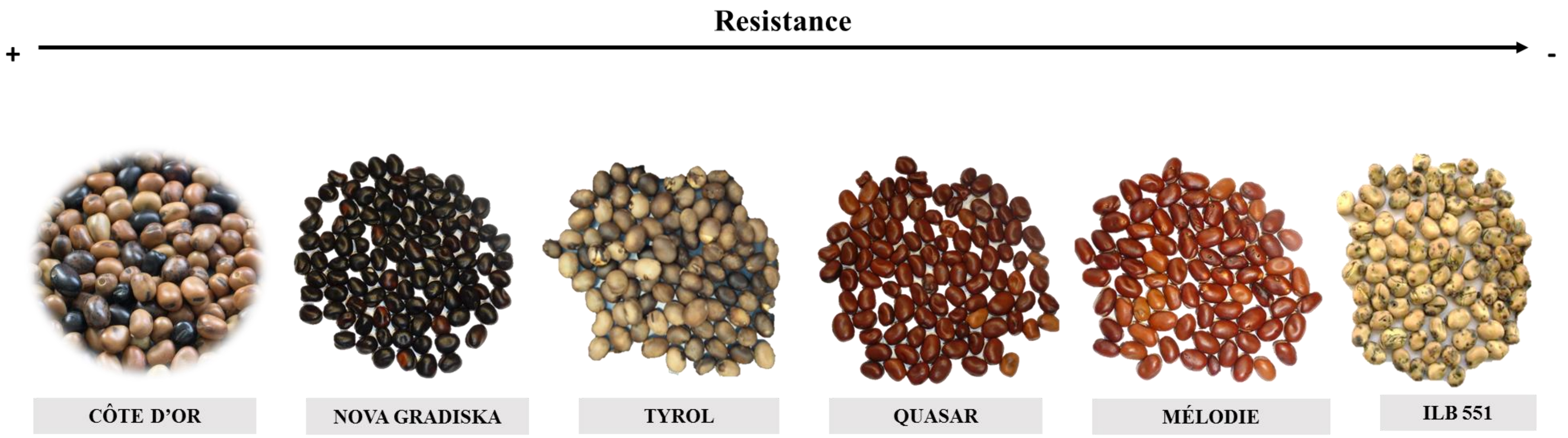

Figure 4. Seeds of different faba bean accessions infested by *Bruchus* spp. representing the gradient of resistance in the germplasm studied. *CÔTE D'OR, NOVA GRADISKA* and *TYROL* were identified among the 10 most resistant accessions in the 4 environments studied, showing a high percentage of healthy seeds (% HS). *QUASAR* exhibited a low percentage of emergence holes (% EH). However, its percentage of surface damage was high (% SD). Cultivar *MÉLODIE* and *ILB 551* were selected as moderately and highly susceptible controls, respectively.
